# The YSQ-R: Predictive Validity and Comparison to the Short and Long Form Young Schema Questionnaire

**DOI:** 10.3390/ijerph20031778

**Published:** 2023-01-18

**Authors:** Ozgur Yalcin, Ida Marais, Christopher William Lee, Helen Correia

**Affiliations:** 1 School of Psychology and Exercise Science, Murdoch University, Murdoch, WA 6150, Australia; 2 Department of Psychiatry, Faculty of Health and Medical Sciences, University of Western Australia, Crawley, WA 6009, Australia; 3 Discipline of Psychological Sciences, Australian College of Applied Professions, Northbridge, WA 6003, Australia

**Keywords:** YSQ-R, YSQ-L3, YSQ-S3, predictive validity, schema, test equating, EMS

## Abstract

The capacity of the Young Schema Questionnaire (YSQ) to predict psychopathology in specific clinical groups has consistently produced mixed findings. This study assessed three versions of the Young Schema Questionnaire (YSQ), including the long form (YSQ-L3), short form (YSQ-S3), and the recent Rasch-derived version, the YSQ-R, and their subscales, in predicting psychological distress in three different psychiatric groups and a non-clinical group. Test equating techniques were first applied to derive a common metric to ensure that each YSQ version was directly comparable. In the second stage, multiple regression analyses were employed to assess the predictive validity of each YSQ version and their subscales. The YSQ-R and YQ-L3 and their respective subscales were similar in their predictive power across all groups and conditions. The YSQ-S3 could not predict pre-treatment Early Maladaptive Schemas (EMS) and global symptom severity in the PTSD group, nor could it predict pre-treatment EMS and changes in global symptom severity in the Alcohol and Substance Use group. This was the first study to assess the predictive validity of three different versions of the YSQ. Our findings suggest that YSQ-R has the breadth of the YSQ-L3 and the shortness of the YSQ-S3, making it an ideal tool for assessing EMS across research and clinical settings.

## 1. Introduction

Schema Therapy (ST) is a treatment model increasingly utilised to treat long-standing and chronic psychological disorders [1,2]. In the model, psychopathology is proposed to develop from unmet childhood needs, particularly secure attachment to others, autonomy and competence, realistic limits and self-control, spontaneity and play, and freedom to express needs and emotions [1,3,4].

Central to ST are constructs known as Early Maladaptive Schemas (EMS) [1,5]. EMS are thought to develop due to adverse childhood experiences such as trauma, abuse, neglect, over or under-protection, and lack of autonomy. If these experiences are protracted, EMS can act as dysfunctional ‘filters’ through which individuals understand themselves, interact with others, and experience the world at large [6,7]. As adults, EMS can interfere with adaptive identity formation, compromise the development of healthy interpersonal relationships, lead to poor emotion regulation skills/capabilities, and thwart academic and professional pursuits. They are mainly unconscious, often leading individuals into situations and towards people who will perpetuate the EMS, inevitably resulting in the needs being left unmet [1,8].

Young et al. [1] proposed 18 EMS in their most recent conceptualisation of EMS within the ST framework, which are grouped into five schema domains that represent the core emotional needs mentioned earlier: Impaired Autonomy and Performance (autonomy and competence), Disconnection and Rejection (secure attachment), Impaired Limits (realistic limits and self-control), Overvigilance and Inhibition (spontaneity and play), and Other-Directedness (freedom to express needs and emotions). More recently, a study by Yalcin et al. [9] proposed a restructure in how some of the EMS are conceptualised and assessed, suggesting 20 rather than 18 EMS (Table 1). Elevated levels of EMS (higher scores indicating a greater level of impairment) consistently emerge across a broad spectrum of psychiatric disorders including post-traumatic stress disorder (PTSD) [10], depression [11,12], obsessive compulsive disorder (OCD) [13], substance use disorders [14], eating disorders [15], and personality disorders [16]. In clinical settings, assessing this transdiagnostic construct is of particular importance. Once identified, EMS can be specifically targeted and corrected with tailored treatment, leading to reductions in other psychopathological symptoms such as depression, anxiety, and interpersonal difficulties [1].

### 1.1. The Young Schema Questionnaire

The assessment of EMS is conducted using the Young Schema Questionnaire (YSQ) [5], which is a self-report scale that has undergone several iterations since its inception, including translations into several languages [17,18,19,20,21,22,23]. As a result of its widespread use, there have been multiple validation studies undertaken on the YSQ; however, investigations of the factor structure, construct, and predictive validity have produced mixed findings. This is likely due to several factors including the use of large nonclinical and small clinical samples, translation effects, different numbers of EMS (and the number of items in them; Table 2), and the exclusive use of factor analytic techniques, when other methods such as Rasch analysis are becoming more commonly employed for questionnaire development and validation [24,25,26].

### 1.2. YSQ-L3 and YSQ-S3

The YSQ-L3 comprises 232 items and was designed to assess 18 EMS [28]. It is recommended for use in clinical settings due to its capacity to capture more subtle nuances in each EMS [29]. Though validation studies on its construct and predictive validity appear sparse, the YSQ-L3 does show some consistency in its predictive validity across various psychological problems.

For example, Cockram, Drummond and Lee [10] hypothesised that EMS severity and symptom severity would decrease following a 12-week outpatient program designed for military veterans with PTSD. They reported that changes in the Impaired Autonomy domain (Dependence, Vulnerability to Harm, Failure, and Enmeshment) were the most significant predictor for symptom change in PTSD, accounting for 26.3% of the variance in change scores. Similar findings were reported by Ahmadian et al. [30], who reported that Vulnerability to Harm, Dependence, Social Isolation and Insufficient Self-control were the biggest predictors of PTSD severity in a cohort of war veterans with either chronic or acute PTSD.

Malogiannis et al. [31] investigated the efficacy of ST in a small sample of chronically depressed outpatients. They reported that changes in the EMS contained in the Impaired Autonomy and Overvigilance and Inhibition domains contributed to the maintenance of reduced depressive and anxiety symptoms over time.

Basile, Tenore, Luppino and Mancini [13] used the YSQ-L3 [28] to explore EMS in 34 outpatients with obsessive compulsive disorder (OCD). Using the Yale–Brown obsessive compulsive scale (Y-BOCS) [32], they found OCD symptom severity was significantly associated with Social Isolation, Failure, Subjugation, and Punitiveness EMS. More specifically, they reported that Social Isolation and Punitiveness predicted OCD severity. In contrast, Kizilagac and Cerit [33] found that Failure and Insufficient Self-Control were most predictive of OCD severity when using the YSQ-S3 in a group of 51 OCD patients.

The YSQ-S3 comprises 90 items and was developed by extracting the five highest correlating items in each of the 18 EMS from the YSQ-L3, and it is purported to be purer factorially [29]. However, there does not appear to be any readily accessible literature detailing its development. Psychometric assessment of the YSQ-S3 has largely focused on the factor structure, reliability, and internal consistency, often with an overreliance on large community samples and smaller clinical groups [20,34,35,36,37]. Nevertheless, predictive and construct validity have been explored in several studies using the YSQ-S3.

A study by Calvete et al. [37] used a student sample and the Spanish YSQ-S3 to investigate how well EMS were associated with psychopathology using the subscales in the SCL-90. They found that depression was primarily associated with Emotional Deprivation, Abandonment, Defectiveness, Social Isolation, Self-Sacrifice, and Negativity. They also reported that anxiety was predicted by Approval-Seeking and Emotional Inhibition. In contrast, Lee et al. [38] reported that Social Isolation, Failure, Mistrust, and Defectiveness predicted depression, and anxiety was best predicted by Social Isolation and Vulnerability to Harm in their student sample.

Wegener et al. [39] assessed whether EMS would predict changes in symptoms in a mixed psychiatric inpatient sample. Using the German YSQ-S3 [27] and GSI in the SCL-90, they found that strong endorsement of the Failure EMS at pre-treatment was the best predictor of EMS and symptom change accounting for 44% of the variance in change scores. Their findings suggest that greater changes in EMS are associated with greater symptom reduction over the course of treatment.

The inconsistencies found in predictive validity for YSQ and its subscales are particularly problematic given the importance of evaluating treatment efficacy and understanding the theoretical underpinnings of the ST model. Even older versions of the YSQ appear to have suffered from the same issues as the more recent iterations.

### 1.3. Previous YSQ Versions

An early study by Stopa [23], the 205-item long form and 75-item short form [5] were comparable and assessed for how well each version predicted psychopathology scores on the GSI in the SCL-90. Using a small sample of psychiatric outpatients, they reported an ‘adequate’ level of internal consistency between the two versions in all but two EMS, Dependence, and Vulnerability to Harm. In the long form, Abandonment, Defectiveness, Subjugation and Unrelenting Standards accounted for 35% of the variance in GSI scores, whereas in the short form it was only Unrelenting Standards that predicted GSI scores, explaining 37% of the variance. In contrast, the study by Nordahl et al. [16] on 104 psychiatric outpatients reported that all 15 EMS in the 205-item Norwegian YSQ [5] predicted symptom change and the GSI in the SCL-90; however, the unique contribution of each EMS varied greatly from 5.1% of the variance for Emotional Deprivation, to 21.1% and 23.7% for Failure and Vulnerability to Harm, respectively.

Renner, Lobbestael, Peeters, Arntz and Huibers [6] investigated the stability of EMS and their relationship with depressive symptoms in a cohort of 132 outpatients with depression. Using the Dutch-translated YSQ-L2 [5], they found that Failure, Abandonment, Emotional Deprivation, and Enmeshment, in that order, significantly predicted depression severity accounting for 48% of the variance. Contrary findings were reported by Welburn et al. [40], who reported that Abandonment and Insufficient Self Control were most predictive of depression in 196 psychiatric outpatients in accord with the 75-item YSQ-SF [25].

More recently, the YSQ-R [9] was proposed as an alternative to the YSQ-L3 and YSQ-S3. The YSQ-R comprises 116 items, measures 20 rather than 18 EMS, and was developed using statistical methods developed based on modern test theory [24]. The use of these techniques may assist in ameliorating the issues mentioned above.

### 1.4. The YSQ-R

Yalcin, Marais, Lee and Correia [9] applied Rasch analysis to the YSQ-L3 using a very large sample (*N* = 838) which included a heterogenous clinical sample (*N* = 575), and a smaller nonclinical group (*N* = 264). They conducted an item-by-item examination of all 232 items based on several statistical fit indicators, including differential item functioning (DIF), item and person fit statistics, local dependence, and item trait fit statistics. Despite removing 116 items due to misfit, the YSQ-R retained excellent reliability across all subscales except for the Enmeshment subscale. Further, the Emotional Inhibition subscale separated into two discrete factors, as did Punitiveness which was confirmed with Principal Components Analyses.

Emotional Inhibition was better conceptualised as Emotional Constriction, which reflects an overcontrol of, and disconnection from emotions, and Fear of Losing Control, which reflects a belief that dire consequences will result from failing to maintain control of emotions. Similarly, the Punitiveness subscale was better represented as Punitiveness (Self), which represents self-directed hypercriticalness, and Punitiveness (Other), which represents the belief that others should be harshly punished for making mistakes [9,41]. These findings have also been found elsewhere [22,41,42]. As such, the YSQ-R captures 20 rather than 18 EMS. The predictive validity of the YSQ-R is yet to be assessed, particularly in comparison to the YSQ-L3 and YSQ-S3.

### 1.5. Aims

This study had two aims. The first was to compare the predictive validity of each YSQ version. The second aim was to investigate the relationship between EMS and symptoms in three different psychiatric groups and a non-clinical group. This included assessing whether baseline EMS severity in the YSQ-L3, YSQ-S3, and YSQ-R could predict baseline symptom severity, and determine whether changes in symptom severity could be predicted by changes in EMS severity across each YSQ version and their subscales. We hypothesised that the YSQ-R would explain more of the variance than the YSQ-S3 and be comparable to the YSQ-L3 at predicting psychological distress, EMS change, and symptom change across all groups.

## 2. Materials and Methods

### 2.1. Participants

Informed consent was obtained from all study participants. Ethics approval was obtained from the relevant University Human Research Ethics Committee and Hospital Ethics Committee. The study was conducted according to the Commonwealth of Australia Privacy Act 1988.

The overall sample (*N* = 684) consisted of a clinical and non-clinical population. All participants in the clinical groups (*N* = 422) were patients attending one of three different day-patient programs in a private psychiatric setting in Western Australia and comprised 206 males and 216 females, with a mean age of 43.06 years (*SD* = 13.70). This sample has also been used elsewhere [9].

### 2.2. Schema Group

This group comprised 35 males and 115 females (*N* = 150), with a mean age of 39.3 years (*SD* = 12.79). Patients in this group primarily had diagnoses of borderline personality disorder, depression, and anxiety as assessed by their referring medical practitioner.

### 2.3. Alcohol and Substance Use Group

This group comprised 87 males and 82 females (*N* = 169), with a mean age of 47.91 years (*SD* = 11.98). The level of dependency and severity were assessed before the commencement of the program using self-report measures. Patients in this group also suffered comorbid depression and anxiety diagnosed by their referring doctor.

### 2.4. PTSD Group

This group comprised 84 males and 19 females (*N* = 103), with a mean age of 43.5 years (*SD* =11.34). All patients were military veterans with PTSD as their primary diagnosis confirmed in accordance with the Clinician-Administered PTSD Scale for DSM-5 (CAPS-V; Weathers et al., 2013) before commencing the program.

### 2.5. Non-Clinical Sample

This sample (*N* = 262) comprised 135 males and 127 females, with a mean age of 35.9 years (*SD* = 12.16). Participants in this sample were recruited from a university participation pool, social media sites including Facebook, and the data collection site Prolific Academic.

### 2.6. Measures

#### 2.6.1. Young Schema Questionnaire—Long Form-3 YSQ-L3 [28]

The YSQ-L3 consists of 232 items measuring 18 different EMS using a six-point Likert scale ranging from 1 “completely untrue” to 6 “describes me perfectly”. Scores are the mean for each EMS subscale, with an average of four or higher indicating that EMS is clinically meaningful [1,43].

#### 2.6.2. Young Schema Questionnaire—Short Form-3 YSQ-S3 [27]

The YSQ-S3 consists of 90 items measuring 18 different EMS using a six-point Likert scale. Similar to the YSQ-L3, scores are the mean for each EMS subscale, with an average of four or higher indicating that EMS is clinically meaningful [1].

#### 2.6.3. Young Schema Questionnaire-Revised YSQ-R [9]

The YSQ-R consists of 116 items measuring 20 different EMS using a six-point Likert scale ranging from “completely untrue of me” to “describes me perfectly”. The YSQ-R contains 20 rather than 18 EMS.

#### 2.6.4. The Symptom Checklist-90-Revised SCL-90-R [44]

The SCL-90-R is a standardised self-report questionnaire consisting of 90-items rated on a 5-point Likert scale and is intended to measure symptom severity across nine sub-scales: Somatisation, Obsessive Compulsive, Interpersonal Sensitivity, Depression, Anxiety, Hostility, Phobic Anxiety, Paranoid Ideation, and Psychoticism. In addition, the Global Severity Index (GSI) is the mean value of all the items and is considered the single best indicator of general psychological distress [45].

#### 2.6.5. Depression, Anxiety and Stress Scale DASS 42 [46]

The DASS-42 is a self-report scale consisting of 42 items and is designed to measure feelings of depression, anxiety, and stress using a four-point Likert scale ranging from “did not apply to me at all” to “applied to me very much, or most of the time”.

#### 2.6.6. PTSD Checklist [47,48]

The PCL is a self-report rating scale designed to assess the DSM symptoms of PTSD using a five-point Likert-type scale ranging from “not at all” to “extremely”. This study utilised the PCL-M (military version) and PCL-5 per the diagnostic criteria found in the DSM-5 (American Psychiatric Association, 2013).

#### 2.6.7. Statistical Considerations and Data Analysis

Data analysis was undertaken in two stages. Although each version of the YSQ measures common latent variables, i.e., schemas, they vary in the YSQ subscales, and the number of items in them. To ensure that the scales in each version were directly comparable, we used a reference metric approach to derive a common metric that expressed the measures on an equal interval scale [49,50]. We adopted a scaling test design and applied test equating techniques to derive the reference metric for each scale using the RUMM2030 software [51,52], with the raw sum scores of the different scales (EMS) as polytomous ‘items’.

In the second stage, we ran multiple regression analyses (MRA) using the statistical package IBM SPSS Statistics (Version 26, IBM Corp., New York, NY, USA) with the Rasch person estimates for each version of the YSQ (which is on the common metric) as independent variables and the outcome measures of each group as dependent variables. Assumptions for all statistical analyses were checked and met. In step one, total YSQ and symptom scores were entered into the model. In the second step, non-significant predictors were removed to explore the unique contribution of each EMS subscale to symptoms.

For the schema group, we had pre- and post-data treatment for the YSQ and SCL-90 [53]. Hence, we could assess the predictive validity of pre-treatment total YSQ and total SCL-90 scores, and we could determine whether changes in each YSQ version could predict changes in the specific subscales of the SCL-90. With the Alcohol and Substance Use group, we had pre-treatment YSQ and pre- and post-DASS 42 [46] data. Therefore, we could assess the predictive validity of each YSQ version at baseline with pre-treatment total DASS-42 scores, and if each YSQ version at baseline could predict symptom change in the DASS-42 and its subscales. As the PTSD group utilised two similar but different versions of the PCL, we calculated z-scores to get a global score of PTSD symptoms, which we then used to assess if pre-treatment YSQ versions could predict pre-treatment global symptom distress. In the non-clinical sample, we assessed the convergent validity of total baseline YSQ and EMS scores and the total scores on the DASS 42 and its subscale.

## 3. Results

Tables S1–S4 in the supplementary tables show the means, standard deviations, and alpha coefficients for the YSQ-R, YSQ-L3, and YSQ-S3, and the clinical and non-clinical groups. Tables S5–S9 in the supplementary tables show regression analyses for pre-treatment total and subscale YSQ and pre-treatment symptoms, and total and subscale YSQ and symptom change in the Schema and Alcohol and Substance Use groups. Effect sizes were calculated using Cohen’s *f*^2^ [54], where a small effect size is ≥*f*^2^ of 0.02, a medium effect size is ≥*f*^2^ of 0.15, and a large effect size ≥ *f*^2^ of 0.35.

### 3.1. Predictive Value of Pre-Treatment EMS and Pre-Treatment Symptom Severity

Table 3 shows results for pre-treatment total YSQ scores and pre-treatment levels of global symptom distress across the different groups.

In the Schema group, symptom scores were assessed using the GSI on the SCL-90 as the dependent variable. The overall regression model was significant for all YSQ versions, YSQ-R (*F* (20, 129) = 8.03, *p* < 0.001), YSQ-L3 (*F* (18, 131) = 9.30, *p* < 0.001), and the YSQ-S3 (*F* (18, 131) = 8.47, *p* < 0.001). For the YSQ-R, global psychological distress was significantly predicted by Social Isolation, Vulnerability to Harm, Enmeshment, Self-Sacrifice, and Failure, which accounted for 51% of the total variance. For the YSQ-L3, the EMS Vulnerability to Harm, Enmeshment, Social Isolation, and Self-Sacrifice accounted for 50% of the variance. For the YSQ-S3, Vulnerability to Harm, Social Isolation, Self-Sacrifice, and Enmeshment explained 50% of the variance in the GSI.

In the Alcohol and Substance Use group, the overall regression model was significant for all YSQ versions, YSQ-R (*F* (20, 148) = 1.78, *p* < 0.05), YSQ-L3 (*F* (18, 150) = 2.48, *p* < 0.001), and the YSQ-S3 (*F* (18, 150) = 1.93, *p* < 0.05). Global psychological distress was uniquely predicted only by Vulnerability to Harm in all YSQ versions accounting for 8% of the unique variance in the YSQ-R, 10.6% of the unique variance in the YSQ-L3, and 11.1% in the YSQ-S3.

In the PTSD group, the overall regression model was significant for the YSQ-R (*F* (20, 82) = 2.35, *p* < 0.05) and the YSQ-L3 (*F* (18, 84) = 2.41, *p* < 0.05), but not the YSQ-S3 (*F* (18, 84) = 1.67, *p* = 0.07). Global psychological distress was uniquely predicted by Vulnerability to Harm, and Dependence, which explained 19% of the variance in the YSQ-R. Similarly, in the YSQ-L3, Vulnerability to Harm, Dependence and Unrelenting Standards explained 22% of the variance. For the YSQ-S3, Unrelenting Standards, and Vulnerability to Harm explained 17% of the variance in global symptom distress.

Symptom scores in the non-clinical group were assessed using the DASS-42 total score as the dependent variable. The overall regression model was non-significant for all YSQ versions, YSQ-R (*F* (19, 242) = 1.14, *p* = 0.31), YSQ-L3 (*F* (19, 240) = 1.07, *p* = 0.38), and the YSQ-S3 (*F* (18, 243) = 0.82, *p* = 0.67). Post hoc visual inspection of the scatter plots revealed no relationships between hence the non-significant findings. Exploration of scatter plots indicated no clear linear pattern.

### 3.2. YSQ Change and Symptom Change

#### 3.2.1. Schema Group

Table 4 shows the results forYSQ and symptom change in the SCL-90 and subscales in the Schema group.

Residualised change scores were calculated for the YSQ total score and the SCL-90 subscales. All changes in all YSQ versions were significantly correlated with changes in total symptom scores (GSI) on the SCL-90. YSQ-R (*F* (20, 128) = 3.76, *p* < 0.001), YSQ-L3 (*F* (18, 131) = 4.11, *p* < 0.001), and the YSQ-S3 (*F* (18, 130) = 3.60, *p* < 0.001). The YSQ-R accounted for more of the variance in GSI change compared to the YSQ-L3 and YSQ-S3. For the YSQ-R, changes to global symptom distress were predicted by changes in Failure, Emotional Constriction, and Social Isolation, which accounted for 34% of the variance in the YSQ-R. Similarly, changes in Failure, Social Isolation, and Emotional Inhibition accounted for 32% of symptom change scores in the YSQ-L3. For the YSQ-S3, changes in Failure, Emotional Inhibition, Defectiveness, and Social Isolation accounted for 30% of the variance in symptom change.

Subsequent subscale analysis of the SCL-90 revealed that all YSQ versions could predict symptom change for seven out of nine subscales except for Somatisation (YSQ-R (*F* (20, 128) = 0.72, *p* = 0.80), YSQ-L3 (*F* (18, 131) = 0.93, *p* = 0.55), YSQ-S3 (*F* (18, 130) = 1.14, *p* = 0.32)) and Phobic Anxiety (YSQ-R (*F* (20, 128) = 1.6, *p* = 0.06), YSQ-L3 (*F* (18, 130) = 1.36, *p* = 0.16), YSQ-S3 (*F* (18, 130) = 1.10, *p* = 0.36)). The YSQ-R was only marginally non-significant in predicting changes in Phobic Anxiety. These findings suggest that changes in schemas co-occur with changes in symptoms. Positive regression coefficients indicate that stronger schema change was associated with greater symptom change.

#### 3.2.2. Alcohol and Substance Use Group

Table 5 shows the results for pre-treatment total YSQ and symptom change scores in the Alcohol and Substance use group.

The overall regression model was significant for the YSQ-R (*F* (20, 148) = 1.67, *p* < 0.05) and the YSQ-L3 (*F* (18, 150) = 2.17, *p* < 0.05), but not the YSQ-S3 (*F* (18, 150) = 1.33, *p* = 0.18). In the YSQ-R, changes to global psychological distress were predicted by Vulnerability to Harm, Punitiveness (Other), and Insufficient Self Control, explaining 11.6% of the variance. Punitiveness (Other) was negatively correlated at baseline, suggesting that lower Punitiveness (Other) is associated with greater change in global symptom severity. In the YSQ-L3, it was only Vulnerability to Harm that explained 5% of the variance, whereas in the YSQ-S3, it was Negativity/Pessimism that explained 4% of the variance in global symptom change.

## 4. Discussion

This is the first study to compare psychometric properties of three different versions of the YSQ. Using three separate psychiatric populations and a non-clinical group, the study had two aims. The first of which was to assess the predictive validity of each YSQ version. The second aim was to assess whether baseline EMS severity in the YSQ-R, YSQ-L3, and YSQ-S3 were predictive of baseline symptom severity and, where possible, determine whether changes in EMS severity across each YSQ version predicted changes in symptom severity.

All versions of the YSQ effectively predicted baseline EMS severity and symptom severity in the Schema and Alcohol and Substance Use groups; however, only the YSQ-R and YSQ-L3 predicted this relationship in the PTSD group. Similarly, only the YSQ-R and YSQ-L3 predicted baseline EMS severity and pre-post symptom changes in the Alcohol and Substance use group. All YSQ versions effectively predicted EMS change and global symptom change in the Schema group. As hypothesised, the YSQ-R explained more of the variance than the YSQ-S3 in predicting global psychological distress, EMS change, and symptom change across all groups, and was directly comparable to the YSQ-L3 in predicting global psychological distress.

### 4.1. Pre-Treatment YSQ Scores and Symptom Severity

In the PTSD group, only the YSQ-R and YSQ-L3 significantly predicted total symptom distress, accounting for 34.1% and 36.4% of the variance, with both showing large effect sizes (0.56 and 0.51, respectively).

Although individual EMS were predictive of symptom severity across all YSQ versions in the PTSD group, there was more consistency in the predictions derived from the YSQ-R and YSQ-L3. For example, Vulnerability to Harm uniquely accounted for the largest variance in the YSQ-R (14.4%) and the YSQL-3 (13.2%) with dependence then accounting for another 4% for each of these measures. In contrast, it was Unrelenting Standards that accounted for the largest portion of the variance (12.3%) in the YSQ-S3, with Vulnerability to Harm explaining an additional 4.7%.

Our findings align with Cockram et al. [10], who found that total YSQ-L3 scores accounted for 22.5% of the variance in PTSD symptoms in war veterans. They also found that Vulnerability to Harm accounted for the strongest association with PTSD symptoms in this cohort. Similarly, Ahmadian et al. [30], reported strong correlations between the YSQ-L3 and PTSD symptoms in veterans with chronic and acute PTSD. The EMS of Vulnerability to Harm, Dependence, Social Isolation, and Insufficient Self-Control were the most predictive of PTSD severity in their study. Given that exposure to life-threatening situations and hypervigilance are two core diagnostic features of PTSD [55], it makes sense from a theoretical and clinical perspective that Vulnerability to Harm, defined by the belief that the world is dangerous, would be the most predictive of PTSD severity in war veterans.

Although EMS have been shown to emerge in a general population sample, none of the YSQ versions in this study predicted psychopathology in the non-clinical group. The YSQ was designed to assess clinical levels of schema severity in clinical populations, hence these findings are consistent with previous literature in the discriminate validity of the YSQ when assessing clinical vs. non-clinical populations [19,38,56].

### 4.2. YSQ, EMS Change, and Symptom Change

#### 4.2.1. Schema Group

Changes in global symptom severity were predicted by changes in EMS severity in all YSQ versions. The YSQ-R was more predictive of changes in EMS and symptom severity in six of the nine subscales of the SCL-90 compared to the YSQ-S3 and had equal to, or greater, predictive power in symptom change scores in five out of nine subscales in the YSQ-L3.

Changes in Failure explained the most variance in global symptom change for all YSQ versions (YSQ-R = 27.7%, YSQ-L3 = 24.6%, and YSQ-S3 = 17.1%). There are two possible explanations for this. First, it may be that specifically targeting a patient’s sense of competence in treatment may increase their sense of self-efficacy and autonomy, leading to greater therapeutic gains in the treatment [57]. Alternatively, there may be other non-specific factors that increase a patient’s sense of agency and autonomy. For example, gaining a better self-understanding (e.g., psychoeducation), may increase their engagement in treatment, leading to greater self-perception as more competent, thereby improving treatment outcomes [58].

Changes in Social Isolation only emerged in the YSQ-R and YSQ-L3 as a statistically significant predictor of symptom change, albeit small (2% and 4%, respectively). This is in line with the recent meta-analysis by Bishop et al. [59], who investigated the role of EMS on depression in adulthood. They found that Social Isolation was the biggest predictor of depression across the 51 included articles. The impact of social isolation and loneliness on mental health is well documented [60].

#### 4.2.2. Alcohol and Substance Use Group

Only the YSQ-R and YSQ-L3 pre-treatment significantly predicted global symptom severity change in the Alcohol and Substance use group, showing moderate effect sizes (0.22 and 0.27, respectively).

For the YSQ-R, higher Vulnerability to Harm, lower Punitiveness (Other), and higher Insufficient Self-Control were most predictive of symptom change; similarly, higher Vulnerability to Harm was most predictive of symptom change in the YSQ-L3. In contrast, only Negativity predicted symptom change in the YSQ-S3. Interestingly, low Punitiveness (Other) and Insufficient Self-Control emerged as predictors of symptom change only in the YSQ-R. This may indicate that greater treatment gains could be obtained by those patients who were generally less persecutory, harsh, and critical of the mistakes of others (perhaps in this case those facilitating and participating in the treatment). Furthermore, Insufficient Self-Control represents difficulty with impulse control which is common in those experiencing substance use problems [61,62].

### 4.3. The Need for Appropriate Item Selection in the Assessment of Latent Variables

As previously mentioned, differences in predicting symptom severity and change between the YSQ-R and YSQ-S3 may be ascribed to differences in item selection. Unlike the YSQ-S3, which was developed by including the five highest correlating items from the YSQ-L3 for each EMS subscale [29], the YSQ-R used Rasch analysis which accounted for item overlap and redundancy and allowed for the inclusion of low-to-high severity items in each subscale, ensuring the breadth and EMS specificity in each schema subscale remained intact.

Secondly, the YSQ-R comprises EMS not contained in the YSQ-S3. For example, there are no items that capture the EMS Fear of Losing Control and Punitiveness (Other) in the YSQ-S3. Additionally, some pertinent and ‘high severity’ items for a given EMS are not included in the YSQ-S3. For example, the item “The world is a dangerous place”, which captures the essence of the Vulnerability to Harm schema, is omitted from the YSQ-S3.

### 4.4. Strengths, Limitations and Future Directions

The heterogeneity of scale-specific metrics considerably impairs the ability to directly compare study outcomes (in our case, YSQ versions and subscales). It has implications for effective communication between researchers and clinicians [25,49]. A unique feature and strength of this study is the use of test equating using the Rasch measurement model, allowing us to create a common metric or “ruler” for the YSQ and its subscales, ensuring that each version was directly comparable by expressing the measures on an equal interval scale [25,49,63,64]. This was a critical first step as although each version of the YSQ measures common latent variables, i.e., EMS, each version is variable in the YSQ subscales, and the number of items contained in them. Test equating methods should be more fastidiously employed where variability across measures assessing common latent variables exists.

Another strength of our study was using three clinical groups and a non-clinical group, contributing to the discussion around specific conditions and their characteristic EMS. This may have implications for designing treatments that are required to reach a broader audience, such as group therapies or self-directed modules.

The current study was limited in its use of a Western sample; the role of EMS in predicting symptoms may differ cross-culturally. For example, while themes such as Failure may be prominent in individualistic and capitalist cultures, themes such as Social Isolation may be more relevant in a collectivist culture. The correlational nature of the current findings also could not determine causality; future research could take a more formulation-driven approach and focus on whether targeted intervention on specific EMS (particularly those in the Impaired Autonomy Domain, which was salient in this study) and EMS-specific to the YSQ-R such as Punitiveness (Others) improves treatment outcomes. Another limitation of this study is that we did not account for possible differences in EMS across demographic groups such as age, gender, socioeconomic status, level of education, employment, and history of treatment. This is important to explore in future studies.

## 5. Conclusions

This was the first study to assess the predictive validity of three different versions of the YSQ, the YSQ-R, YSQ-L3, and YSQ-S3. The YSQ-R and YSQ-L3 and their respective subscales were similar in their predictive power across all groups and conditions. In contrast, the YSQ-S3 could not predict pre-treatment EMS and global symptom severity in the PTSD group, nor could it predict pre-treatment EMS and changes in global symptom severity in the Alcohol and Substance use group. The shortfalls of the YSQ-S3 may be attributable to several factors including, but not limited to, poor item selection, the fact that two entire EMS are not included, i.e., Fear of Losing Control and Punitiveness (Others), and the exclusive use of factor analytic techniques. Hence, the YSQ-R has the breadth of the YSQ-L3 and the shortness of the YSQ-S3, making it an ideal tool for assessing EMS across research and clinical settings. More research is required to establish the YSQ-R as the preferred EMS assessment tool, including its cross-cultural application, and across different diagnostic groups.

## Figures and Tables

**Table 1:** Eighteen Early Maladaptive Schemas (EMS) and Associated Themes as Proposed by Young et al. [1].

	EMS	Associated Themes
Disconnection/Rejection	Abandonment	The expectation that important others will eventually abandon them
	Mistrust/Abuse	The expectation that others will intentionally abuse, and manipulate them
	Emotional Deprivation	The expectation that others will not adequately meet one’s needs for nurturance and support
	Defectiveness/Shame	The belief that one is fundamentally flawed, defective, or unlovable
	Social Isolation	The feeling of not belonging or fitting into society
Impaired Autonomy	Dependence	The feeling that one is completely hopeless, dependant on others and is incapable of making everyday decisions
	Vulnerability to Harm	The belief that the world is dangerous, and that disaster can strike at any moment
	Enmeshment	Excessive emotional involvement with others (usually parents) due to the belief that one cannot cope without the other
	Failure	The belief that one is fundamentally inadequate compared to others
Impaired Limits	Entitlement	The belief that one is superior to others and is entitled to special privileges and rights
	Insufficient Self-Control	Difficulties exercising self-control to achieve goals, low frustration tolerance, and inability to control urges and impulses
Other-Directedness	Subjugation	Excessive subjugation of needs to avoid punishment, abandonment, and rejection.
	Self-Sacrifice	Excessive or exaggerated expectations of duty to meet the needs of others at the expense of own fulfilment
	Approval Seeking	Excessive focus on gaining the attention, recognition, and approval of others often at the expense one’s own sense of self
Overvigilance and Inhibition	Negativity	An increased focus on the negative aspects of life, whist minimising and neglecting the positive
	* Emotional Inhibition	The belief that the expression of emotion may lead to negative consequences such as harm or embarrassment to others
	Unrelenting Standards	The belief that one will be harshly criticised if they do not meet very high standards (often internalised standards) of performance or behaviour
	* Punitiveness	The belief that people, including themselves, who make mistakes should be harshly punished
EMS from YSQ-R yet to be assigned to domains	† Emotional Constriction	Excessive overcontrol of emotions due to feelings of shame and embarrassment of all emotions
	† Fear of Losing Control	A belief that dire consequences will result from failing to maintain control of emotions
	† Punitiveness (Self)	Self-directed hyper-criticalness and punishment for a person’s own mistakes, suffering, or imperfections
	† Punitiveness (Other)	The belief that others should be harshly punished and suffer consequences for indiscretions and mistakes

Note: † *=* EMS in the YSQ-R; * = EMS not contained in the YSQ-R. Add YSQ-R reference.

**Table 2:** Number of Items and Early Maladaptive Schemas (EMS) in the Various Versions of the Young Schema Questionnaire (YSQ).

YSQ	Number of Items	Number of EMS
YSQ [5]	205	* 16
YSQ-L2 [5]	205	15
YSQ-SF [25]	75	15
YSQ-L3 [1]	232	^ 18
YSQ-S3 [27]	90	18
YSQ-R [9]	116	† 20

Note: * = Social Undesirability removed from YSQ. ^ = Approval Seeking, Negativity/Pessimism, and Punitiveness were added. † = Emotional Inhibition and Punitiveness were replaced by Fear of Losing Control, Emotional Constriction, Punitiveness (Self), and Punitiveness (Other).

**Table 3:** Regression Analysis of Total Pre-Treatment EMS in each YSQ Version and Pre-Treatment Total Symptom Scores Across Each Group.

	YSQ-R		YSQ-L3		YSQ-S3	
Group	*R* ^2^	*f* ^2^	*R* ^2^	*f* ^2^	*R* ^2^	*f* ^2^
Schema	0.55 **	1.22	0.56 **	1.27	0.53 **	1.12
Alcohol	0.19 *	0.23	0.23 **	0.29	0.18 *	0.21
PTSD	0.36 **	0.56	0.34 **	0.51	0.26	**-**
Non-Clinical	0.08	**-**	0.07	-	0.05	**-**

Note. * = Correlation is significant at the 0.05 level (2-tailed). ** = Correlation is significant at the 0.001 level (2-tailed). *f*^2^ *=* Cohens *f* where a small effect size is ≥0.02, medium is ≥0.15, and a large is ≥0.35.

**Table 4:** Regression Analysis for Schema Change and Symptom Change for Each YSQ Version and SCL-90 Subscales in the Schema Group.

Predictors	YSQ-R		YSQ-L3		YSQ-S3	
SCL-90 Subscales	*R* ^2^	*f* ^2^	*R* ^2^	*f* ^2^	*R* ^2^	*f* ^2^
GSI	0.37 *	0.59	0.36 *	0.53	0.33 *	0.49
Somatisation	0.10	-	0.11	-	0.14	-
Obsessive/Compulsive	0.34 *	0.55	0.30 *	0.43	0.32 *	0.47
Interpersonal Sensitivity	0.44 *	0.79	0.41 *	0.70	0.34 *	0.55
Depression	0.32 *	0.47	0.33 *	0.49	0.28 *	0.40
Anxiety	0.30 *	0.43	0.25 **	0.33	0.29 *	0.41
Hostility	0.26 *	0.35	0.27 *	0.37	0.27 *	0.37
Phobic anxiety	0.20	-	0.15	-	0.13	-
Paranoid ideation	0.28 *	0.39	0.26 *	0.35	0.23 **	0.30
Psychoticism	0.33 *	0.49	0.33 *	0.49	0.31 *	0.45

Note. * = Correlation is significant at the 0.05 level (2-tailed). ** = Correlation is significant at the 0.001 level (2-tailed). *f*^2^ = Cohens *F*, GSI = Global Severity Index.

**Table 5:** Regression Analysis for total Pre-Treatment YSQ and Subscale Symptom Change in the Alcohol and Substance Use Group.

	YSQ-R		YSQ-L3		YSQ-S3	
Predictors	*R* ^2^	*f* ^2^	*R* ^2^	*f* ^2^	*R* ^2^	*f* ^2^
DASS-42 (total)	18 **	0.22	0.21 **	0.25	0.14	-
Depression	0.21 **	0.27	0.21 **	0.27	0.16	-
Anxiety	0.15	-	0.19 **	0.23	0.13	-
Stress	0.15	-	0.18 **	0.22	0.12	-

Note. ** = Correlation is significant at the 0.001 level (2-tailed). *f*² = Cohens *F.*

## Data Availability

Data from this study can be made available upon reasonable request from the lead author.

## Supplementary Materials

The following supporting information can be downloaded at: https://www.mdpi.com/article/10.3390/ijerph20031778/s1, Table S1: Alpha Coefficients, Means and Standard Deviations for Early Maladaptive Schemas (EMS) for the Schema Group (*n* = 150); Table S2: Alpha Coefficients, Means and Standard Deviations for Early Maladaptive Schemas (EMS) for the PTSD group (*n* = 103); Table S3: Alpha Coefficients, Means and Standard Deviations for Early Maladaptive Schemas (EMS) for the Alcohol group (*n* = 169); Table S4: Alpha Coefficients, Means and Standard Deviations for Early Maladaptive Schemas (EMS) for the Non-Clinical Group (*n* = 264); Table S5: Regression Analysis for Pre-Treatment YSQ and Pre-Treatment Symptoms Across Each Group; Table S6: Regression Analysis for Schema Change and Symptom Change for the YSQ-R and SCL-90 Subscales in the Schema Group; Table S7: Regression Analysis for Schema Change and Symptom Change for the YSQ-L3 and SCL-90 Subscales in the Schema Group; Table S8: Regression Analysis for Schema Change and Symptom Change for the YSQ-S3 and SCL-90 Subscales in the Schema Group; Table S9: Regression Analysis for Pre-Treatment YSQ Change and Symptom Change for Each YSQ Version and DASS-42 Subscales in the Alcohol and Substance Use Group.
