# Supplementary material for: The YSQ-R: Predictive Validity and Comparison to the Short and Long Form Young Schema Questionnaire
Source: Int J Environ Res Public Health. 2023 Jan 18;20(3):1778. doi: 10.3390/ijerph20031778 (PMC9914719; doi:10.3390/ijerph20031778)
Supplement: Supplementary file 1 [file ijerph-20-01778-s001.zip › ijerph-2099407-supplementary.pdf]

Supplementary Table S1

Alpha Coefficients, Means and Standard Deviations for Early Maladaptive Schemas (EMS) for the Schema Group (*n*= 150)

(EMS)	YSQ-L3		YSQ-S3		YSQ-R	
	Alpha (*α*)	Means (*SD*)	Alpha (*α*)	Means (*SD*)	Alpha (*α*)	Means (*SD*)
Emotional Deprivation	.95	3.66 (1.36)	.92	3.71 (1.44)	.91	3.60 (1.38)
Abandonment	.93	3.39 (1.16)	.90	3.41 (1.44)	.88	3.33 (1.20)
Mistrust/Abuse	.93	3.16 (1.09)	.90	3.18 (1.34)	.80	2.81 (1.15)
Social Isolation	.91	3.83 (1.20)	.93	4.06 (1.38)	.88	3.84 (1.32)
Defectiveness	.95	3.40 (1.23)	.91	3.49 (1.44)	.90	3.57 (1.33)
Failure	.95	3.77 (1.40)	.94	3.57 (1.54)	.93	3.86 (1.43)
Dependence/Incompetence	.95	3.08 (1.21)	.85	2.80 (1.21)	.92	3.23 (1.27)
Vulnerability to Harm	.89	2.77 (1.04)	.78	2.53 (1.13)	.79	2.54 (1.13)
Enmeshment	.90	2.28 (1.10)	.80	2.21 (1.16)	.85	2.24 (1.11)
Subjugation	.92	3.47 (1.20)	.89	3.51 (1.31)	.85	3.53 (1.26)
Self-Sacrifice	.93	3.97 (1.07)	.92	3.69 (1.19)	.86	4.02 (1.22)
Emotional Inhibition	.84	2.97 (1.03)	.85	2.77 (1.18)	.82	-
Unrelenting Standards	.93	3.62 (1.12)	.84	4.11 (1.21)	.88	3.65 (1.25)
Entitlement	.90	2.32 (.99)	.86	2.21 (1.09)	.82	2.42 (1.08)
Insufficient Self Control	.91	3.23 (1.04)	.86	3.45 (1.24)	.85	3.31 (1.13)
Approval Seeking	.93	3.31 (1.13)	.87	2.97 (1.27)	.88	3.65 (1.30)
Negativity/Pessimism	.92	3.65 (1.22)	.90	3.59 (1.43)	.87	3.50 (1.28)
Punitiveness	.91	3.35 (1.04)	.92	3.31 (1.24)	.92	-
Fear of Losing Control	-	-	-	-	.82	3.21 (1.30)
Emotional Constriction	-	-	-	-	.85	2.77 (1.18)
Punitiveness (Self)	-	-	-	-	.93	3.22 (1.44)
Punitiveness (Other)	-	-	-	-	.79	2.92 (1.11)

Supplementary Table S2

Alpha Coefficients, Means and Standard Deviations for Early Maladaptive Schemas (EMS) for the PTSD group (*n*= 103)

(EMS)	YSQ-L3		YSQ-S3		YSQ-R	
	Alpha (*α*)	Means (*SD*)	Alpha (*α*)	Means (*SD*)	Alpha (*α*)	Means (*SD*)
Emotional Deprivation	.92	3.16 (1.26)	.89	3.16 (1.33)	.85	3.1 (1.27)
Abandonment	.92	2.85 (1.13)	.89	2.74 (1.39)	.85	2.76 (1.17)
Mistrust/Abuse	.94	3.33 (1.16)	.88	3.44 (1.38)	.83	3.13 (1.29)
Social Isolation	.93	3.58 (1.31)	.94	3.92 (1.47)	.88	3.67 (1.35)
Defectiveness	.95	2.95 (1.29)	.93	2.99 (1.52)	.92	2.98 (1.45)
Failure	.94	2.85 (1.4)	.92	2.66 (1.41)	.92	2.88 (1.43)
Dependence/Incompetence	.94	2.62 (1.13)	.77	2.45 (1.1)	.89	2.68 (1.21)
Vulnerability to Harm	.91	2.86 (1.31)	.89	2.68 (1.36)	.90	2.66 (1.41)
Enmeshment	.91	2.4 (1.06)	.75	2.57 (1.08)	.84	2.56 (1.1)
Subjugation	.87	3.06 (1.15)	.82	3.11 (1.28)	.72	2.78 (1.12)
Self-Sacrifice	.89	2.1 (0.81)	.75	1.66 (0.93)	.64	2.22 (0.85)
Emotional Inhibition	.85	2.99 (1.07)	.75	3.31 (1.15)	-	-
Unrelenting Standards	.85	3.62 (0.88)	.60	3.51 (0.99)	.79	3.8 (1.07)
Entitlement	.85	3.74 (1.15)	.83	3.92 (1.35)	.78	3.68 (1.21)
Insufficient Self Control	.90	3.45 (1.07)	.79	3.66 (1.23)	.86	3.32 (1.23)
Approval Seeking	.88	2.86 (0.98)	.70	2.93 (1.1)	.77	2.85 (1.12)
Negativity/Pessimism	.86	3.44 (1.07)	.75	3.72 (1.14)	.75	3.51 (1.09)
Punitiveness	.92	2.58 (1.09)	.84	3.01 (1.29)	-	-
Fear of Losing Control	-	-	-	-	.84	2.55 (1.26)
Emotional Constriction	-	-	-	-	.71	3.02 (1.12)
Punitiveness (Self)	-	-	-	-	.84	2.67 (1.26)
Punitiveness (Other)	-	-	-	-	.66	2.72 (1.06)

Supplementary Table S3

Alpha Coefficients, Means and Standard Deviations for Early Maladaptive Schemas (EMS) for the Alcohol group (*n*= 169)

(EMS)	YSQ-L3		YSQ-S3		YSQ-R	
	Alpha (*α*)	Means (*SD*)	Alpha (*α*)	Means (*SD*)	Alpha (*α*)	Means (*SD*)
Emotional Deprivation	.95	3.15 (1.42)	.91	3.17 (1.48)	.90	3.07 (1.44)
Abandonment	.91	2.83 (1.07)	.87	3.01 (1.37)	.82	2.71 (1.08)
Mistrust/Abuse	.92	2.85 (1.1)	86	2.86 (1.31)	.80	2.49 (1.15)
Social Isolation	.93	3.02 (1.36)	.93	3.13 (1.53)	.90	3.09 (1.46)
Defectiveness	.94	2.68 (1.19)	.87	3.10 (1.19)	.90	2.60 (1.31)
Failure	.94	2.71 (1.36)	.93	2.52 (1.43)	.92	2.75 (1.39)
Dependence/Incompetence	.93	2.5 (1.11)	.75	2.35 (1.04)	.90	2.60 (1.20)
Vulnerability to Harm	.88	2.59 (1.03)	.77	2.47 (1.15)	.70	2.5 (1.05)
Enmeshment	.89	2.05 (1.03)	.84	2.00 (1.20)	.82	2.06 (1.04)
Subjugation	.88	3.05 (1.14)	.82	2.98 (1.24)	.79	3.16 (1.21)
Self-Sacrifice	.91	3.81 (1.03)	.82	3.63 (1.25)	.85	3.77 (1.19)
Emotional Inhibition	.86	2.9 (1.16)	.88	2.64 (1.36)	-	-
Unrelenting Standards	.90	3.45 (1.07)	.78	3.79 (1.19)	.82	3.42 (1.15)
Entitlement	.87	2.71 (1.03)	.81	2.77 (1.18)	.78	2.85 (1.10)
Insufficient Self Control	.90	3.45 (1.04)	.83	3.49 (1.28)	.83	3.57 (1.16)
Approval Seeking	.91	3.09 (1.09)	.85	2.99 (1.21)	.82	3.18 (1.26)
Negativity/Pessimism	.90	3.06 1.17)	.86	3.14 (1.33)	.82	2.92 (1.19)
Punitiveness	.90	3.17 (1.05)	.83	2.84 (1.25)	-	-
Fear of Losing Control		-		-	.79	3.22 (1.34)
Emotional Constriction		-		-	.88	2.64 (1.36)
Punitiveness (Self)		-		-	.83	2.88 (1.24)
Punitiveness (Other)		-		-	.78	3.08 (1.18)

Supplementary Table S4

Alpha Coefficients, Means and Standard Deviations for Early Maladaptive Schemas (EMS) for the Non-Clinical Group (*n*= 264)

(EMS)	YSQ-L3		YSQ-S3		YSQ-R	
	Alpha (*α*)	Means (*SD*)	Alpha (*α*)	Means (*SD*)	Alpha (*α*)	Means (*SD*)
Emotional Deprivation	.95	2.78 (1.33)	.93	2.80 (1.45)	.91	2.75 (1.36)
Abandonment	.95	2.34 (1.09)	.90	2.30 (1.25)	.90	2.20 (1.11)
Mistrust/Abuse	.94	2.67 (1.07)	.89	2.81 (1.25)	.85	2.39 (1.11)
Social Isolation	.94	2.78 (1.24)	.93	3.05 (1.39)	.90	2.86 (1.30)
Defectiveness	.96	2.19 (1.20)	.89	2.65 (1.28)	.95	2.22 (1.33)
Failure	.95	2.25 (1.21)	.94	2.18 (1.26)	.93	2.32 (1.22)
Dependence/Incompetence	.96	2.01 (1.07)	.84	1.90 (1.01)	.93	2.06 (1.12)
Vulnerability to Harm	.94	2.38 (1.14)	.88	2.24 (1.20)	.84	2.40 (1.14)
Enmeshment	.93	1.76 (.94)	.85	1.78 (0.99)	.89	1.78 (.97)
Subjugation	.93	2.31 (1.09)	.90	2.22 (1.13)	.86	2.34 (1.12)
Self-Sacrifice	.93	3.42 (1.01)	.84	3.27 (1.19)	.86	3.38 (1.16)
Emotional Inhibition	.89	2.54 (1.13)	.90	2.71 (1.36)	-	-
Unrelenting Standards	.93	3.02 (1.08)	.82	3.40 (1.17)	.88	3.09 (1.06)
Entitlement	.89	2.35 (.93)	.81	2.31 (1.01)	.80	2.41 (0.97)
Insufficient Self Control	.93	2.44 (1.00)	.87	2.57 (1.16)	.87	2.55 (1.07)
Approval Seeking	.95	2.5 (1.13)	.88	2.52 (1.14)	.89	2.57 (1.16)
Negativity/Pessimism	.95	2.98 (1.07)	.92	3.12 (1.39)	.91	2.97 (1.29)
Punitiveness	.93	2.92 (1.04)	.86	2.73 (1.13)	-	-
Fear of Losing Control	-	-		-	.86	2.32 (1.25)
Emotional Constriction	-	-		-	.91	2.71 (1.36)
Punitiveness (Self)	-	-		-	.85	2.67 (1.09)
Punitiveness (Other)	-	-		-	.81	2.99 (1.16)

Supplementary Table S5
Regression Analysis for Pre-Treatment YSQ and Pre-Treatment Symptoms Across Each Group

	Predictor	*B*	SE	β	*t*	*F*	R^2	f^2	VIF
				Schema Group					
Step 1									
	**(YSQ-R Total)**	-	-	-	-	8.03	.55**	1.22	-
Step 2									
	Social Isolation	.214	.065	.227	3.29	-	.26**	.35	1.39
	Vulnerability to Harm	.195	.055	.232	3.54	-	.12**	.14	1.26
	Enmeshment	.190	.050	.238	3.78	-	.08**	.09	1.16
	Self-Sacrifice	.231	.076	.193	3.03	-	.03*	.03	1.19
	Failure	.214	.080	.185	2.68	-	.24*	.03	1.39
Step 1									
	**(YSQ-L3 Total)**	-	-	-	-	9.30*	.56**	1.27	-
Step 2									
	Vulnerability to Harm	.308	.067	.319	4.60	-	.32**	.47	1.38
	Enmeshment	.209	.052	.252	4.03	-	.09**	.10	1.14
	Social Isolation	.217	.067	.225	3.26	-	.05**	.05	1.38
	Self-Sacrifice	.275	.094	.195	2.94	-	.03*	.03	1.28
Step 1									
	**(YSQ-S3 Total)**	-	-	-	-	8.47*	.54**	1.17	-
Step 2									
	Vulnerability to Harm	.333	.058	.377	5.71	-	.32**	.47	1.21
	Social Isolation	.259	.066	.255	3.92	-	.09**	.10	1.97
	Self-Sacrifice	.236	.073	.207	3.26	-	.03*	.03	1.14
	Enmeshment	.150	.048	.196	3.13	-	.03*	.03	1.06
				Alcohol Group					
	Predictor	*B*	SE	β	*t*	*F*	R^2	f^2	VIF
Step 1									
	**(YSQ-R total)**	-	-	-	-	1.78**	.19	.23	-
Step 2									
	Vulnerability to Harm	.077	.020	.283	3.81*	-	.08	.09	1.00
Step 1									
	**(YSQ-L3 total)**	-	-	-	-	2.48*	.23	.30	-
Step 2									
	Vulnerability to Harm	.090	.020	.326	4.45*	-	.11	.12	1.00
Step 1									
	**(YSQ-S3 total)**	-	-	-	-	1.93**	.19	.23	--
Step 2									
	Vulnerability to Harm	.091	.020	.334	4.58*	-	.11	.12	1.00
				PTSD Group					
	Predictor	*B*	SE	β	*t*	*F*	R^2	f^2	VIF
Step 1									
	**(YSQ-R total)**	-	-	-	-	2.35*	.36*	.56	-
Step 2									
	Vulnerability to Harm	.118	.034	.321	3.43	-	14**	.16	1.08
	Dependence	.085	.037	.217	2.31	-	.04*	.04	1.08
Step 1									
	**(YSQ-L3 total)**	-	-	-	-	2.41*	.34*	.52	-
Step 2									
	Vulnerability to Harm	.078	.038	.209	2.04	-	.13**	.15	1.33
	Dependence	.086	.036	.221	2.40	-	.04*	.04	1.06
	Unrelenting Standards	.212	.097	.219	2.20	-	.04*	.04	1.26
Step 1									
	**(YSQ-S3 total)**	-	-	-	-	1.64	.26	.35	-
Step 2									
	Unrelenting Standards	.229	.094	.247	2.45	-	.12**	.14	1.23
	Vulnerability to Harm	.089	.037	.241	2.39	-	.05*	.05	1.23
				Non-Clinical Group					
	Predictor	*B*	SE	β	*t*	*F*	R^2	f^2	VIF
Step 1									

	**(YSQ-R total)**	-	-	-	-	1.13	.08	.09	
Step 2		-	-	-	-	-	-	-	
Step 1	**(YSQ-L3 total)**	-	-	-	-	1.07	.08	.09	
Step 2		-	-	-	-	-	-	-	
Step 1	**(YSQ-S3 total)**	-	-	-	-	.82	.06	.06	
Step 2		-	-	-	-	-	-	-	

Note. *= Correlation is significant at the 0.01 level (2-tailed). **= Correlation is significant at the 0.05 level (2-tailed). YSQ = Young Schema Questionnaire. *B*= Unstandardised Coefficient, SE=Standard Error, β=Standardised Beta Coefficient, R^2=R squared. f^2 = Cohens *f*, VIF = Variance Inflation Factor where a VIF of 1 = No correlation, < 3 = low correlation, > 5 = high correlation).

Supplementary Table S6

Regression Analysis for Schema Change and Symptom Change for the YSQ-R and SCL-90 Subscales in the Schema Group

		**YSQ-R**							
SCL-90 Subscales		*B*	SE	β	*t*	*F*	R^2 (Change)	f^2	VIF
Step 1									
	**GSI**	-	-	-	-	3.76	.37*	.59	-
Step 2									
	Failure	.380	.083	.376	4.60	-	.28**	.39	1.45
	Emotional Constriction	.238	.095	.183	2.49	-	.04*	.04	1.72
	Social Isolation	.163	.079	.169	2.06	-	.02*	.02	1.48
Step 1									
	**Somatisation**	-	-	-	-	*.72*	*.10*	*.11*	-
Step 2									
	Punitiveness (Other)	*.382*	.151	.204	2.53	-	*.04**	.04	1.00
Step 1									
	**Obsessive/Compulsive**	-	-	-	-	3.25	.34**	.52	-
Step 2									
	Dependence	.495	.144	.306	3.44	-	.21**	.27	1.55
	Failure	.333	.115	.258	2.89	-	.04*	.04	1.55
Step 1									
	**Interpersonal Sensitivity**	-	-	-	-	4.99	.44**	.79	-
Step 2									
	Failure	.464	.125	.304	3.72	-	.29**	.41	1.59
	Social Isolation	.295	.114	.203	2.57	-	.04*	.04	1.48
	Enmeshment	.278	.094	.206	2.95	-	.04*	.04	1.60
	Emotional Constriction	.305	.138	.156	2.21	-	.02*	.02	1.18
Step 1									
	**Depression**	-	-	-	-	3.06	.32**	.47	-
Step 2									
	Failure	.375	.159	.229	2.37	-	.20**	.25	1.85
	Social Isolation	.300	.133	.193	2.51	-	.03*	.03	1.44
	Dependence	.405	.184	.198	2.20	-	.02*	.02	1.56
Step 1									
	**Anxiety**	-	-	-	-	2.74	.30**	.43	-
Step 2									
	Failure	.481	.107	.355	4.49	-	.15*	.18	1.16
	Emotional Constriction	.459	.139	.263	3.29	-	.04*	.04	1.18
	Emotional Deprivation	-.178	.085	-.165	-2.10	-	.02*	.02	1.15
Step 1									
	**Hostility**	-	-	-	-	2.49	.26*	.35	-
Step 2									
	Failure	.468	.111	.335	4.23	-	.15**	.18	1.11
	Emotional Constriction	.314	.142	.174	2.20	-	.03*	.03	1.11

Step 1									
	**Phobic anxiety**	-	-	-	-	*1.59*	*.20*	*.25*	-
Step 2									
	Social Isolation	.258	.106	.208	2.43	-	.08**	.09	1.21
	Dependence	.315	.139	.194	2.26	-	.03*	.03	1.21
Step 1									
	**Paranoid ideation**	-	-	-	-	2.44	.28**	.39	-
Step 2									
	Failure	.713	.114	.458	6.26	-	.21**	.27	1.00
Step 1									
	**Psychoticism**	-	-	-	-	3.12	.33**	.49	-
Step 2									
	Failure	.306	.091	.285	3.36	-	.19**	.23	1.40
	Abandonment	.299	.125	.200	2.39	-	.04*	.04	1.37
	Emotional Constriction	.235	.106	.170	2.22	-	.03*	.03	1.14

Note. *= Correlation is significant at the 0.01 level (2-tailed). **= Correlation is significant at the 0.05 level (2-tailed). YSQ = Young Schema Questionnaire. *B*= Unstandardised Coefficient, SE=Standard Error, β=Standardised Beta Coefficient, R^2=R squared, f^2 = Cohens *f*. GSI = Global Severity Index. VIF = Variance Inflation Factor where a VIF of 1 = No correlation, < 3 = low correlation, > 5 = high correlation).

Supplementary Table S7
Regression Analysis for Schema Change and Symptom Change for the YSQ-L3 and SCL-90 Subscales in the Schema Group

				YSQ-L3					
SCL-90	Subscales	*B*	SE	β	*t*	*F*	R^2 (Change)	f^2	VIF
Step 1									
	**GSI**	-	-	-	-	4.11	.36**	.56	-
Step 2									
	Failure	.315	.095	.291	3.17	-	.25**	.33	1.63
	Social Isolation	.236	.086	.224	2.73	-	.04*	.04	1.43
	Emotional Inhibition	.263	.113	.180	2.32	-	.02*	.02	1.27
Step 1									
	**Somatisation**	-	-	-	-	*.93*	*.11*	-	-
Step 2									
	Punitiveness	.364	.154	.191	2.36	-	.04*	.04	1.00
Step 1									
	**Obsessive/Compulsive**	-	-	-	-	3.13	.30**	.43	-
Step 2									
	Dependence	.531	.154	.301	3.46	-	.21**	.27	1.49
	Punitiveness	.478	.157	.265	3.04	-	.05*	.05	1.49
Step 1									
	**Interpersonal Sensitivity**	-	-	-	-	5.19	.41**	.69	-
Step 2									
	Failure	.393	.139	.242	2.82	-	.27**	.37	1.72
	Social Isolation	.388	.124	.245	3.13	-	.05**	.05	1.43
	Enmeshment	.252	.102	.180	2.48	-	.04*	.04	1.23
	Emotional Constriction	.352	.165	.161	2.13	-	.02*	.02	1.32
Step 1									
	**Depression**	-	-	-	-	3.60	.33**	.49	-
Step 2									
	Social Isolation	.589	.134	.344	4.38	-	.20**	.25	1.24
	Punitiveness	.472	.188	.205	2.52	-	.05**	.05	1.34
	Entitlement	.231	.099	.172	2.35	-	.03*	.03	1.09
Step 1									
	**Anxiety**	-	-	-	-	2.39	.25*	.33	-
Step 2									
	Dependence	.470	.182	.254	2.59	-	.15**	.18	1.71
	Failure	.301	.142	.208	2.12	-	.02*	.02	1.71
Step 1									
	**Hostility**	-	-	-	-	2.74	.27**	.37	-

Step 2									
	Failure	.264	.144	.177	1.83	-	.15**	.18	1.73
	Defectiveness	.392	.178	.206	2.21	-	.04*	.04	1.60
	Emotional Inhibition	.361	.169	.179	2.14	-	.02*	.02	1.29
Step 1									
	**Phobic anxiety**	-	-	-	-	*1.36*	*.16*	-	-
Step 2									
	Dependence	.499	.139	.282	3.58	-	.08**	.09	1.00
	**Paranoid ideation**	-	-	-	-	2.55	.26**	.35	-
Step 2									
	Failure	.542	.138	.327	3.93	-	.17**	.20	1.27
	Emotional Inhibition	.415	.186	.186	2.23	-	.03*	.03	1.27
Step 1									
	**Psychoticism**	-	-	-	-	3.62	.33**	.49	-
Step 2									
	Punitiveness	.449	.120	.299	3.73	-	.19**	.23	1.26
	Defectiveness	.428	.118	.291	3.63	-	.07**	.08	1.26

Note. *= Correlation is significant at the 0.01 level (2-tailed). **= Correlation is significant at the 0.05 level (2-tailed). YSQ = Young Schema Questionnaire. *B*= Unstandardised Coefficient, SE=Standard Error, β=Standardised Beta Coefficient, R^2=R squared, f^2 = Cohens *f*. GSI = Global Severity Index. VIF = Variance Inflation Factor where a VIF of 1 = No correlation, < 3 = low correlation, > 5 = high correlation).

Supplementary Table S8

Regression Analysis for Schema Change and Symptom Change for the YSQ-S3 and SCL-90 Subscales in the Schema Group

				YSQ-S3					
SCL-90	Subscales	*B*	SE	β	*t*	*F*	R^2 (Change)	f^2	VIF
Step 1									
	**GSI**	-	-	-	-	3.60	.33**	.49	-
Step 2									
	Failure	.199	.083	.199	2.40	-	.17**	.20	1.42
	Emotional Inhibition	.271	.097	.208	2.80	-	.07**	.08	1.14
	Defectiveness	.235	.093	.206	2.52	-	.04*	.04	1.38
	Self-Sacrifice	.208	.090	.176	2.32	-	.03*	.03	1.19
Step 1									
	**Somatisation**	-	-	-	-	1.14	.14	.16	-
Step 2									
	Vulnerability to Harm	.348	.107	.313	3.26	-	.04*	.04	1.44
	Negativity	-.289	.139	-.199	-2.07	-	.03*	.03	1.44
Step 1									
	**Obsessive/Compulsive**	-	-	-	-	3.37	.32**	.47	-
Step 2									
	Dependence	.436	.107	.325	4.09	-	.19**	.23	1.23
	Mistrust/Abuse	.256	.110	.181	2.32	-	.04*	.04	1.17
	Self-Sacrifice	.234	.118	.155	1.98	-	.02*	.02	1.83
Step 1									
	**Interpersonal Sensitivity**	-	-	-	-	3.71	.34**	.52	-
Step 2									
	Subjugation	.373	.134	.231	2.79	-	.21**	.27	1.47
	Social Isolation	.278	.126	.178	2.21	-	.07**	.08	1.37
	Emotional Inhibition	.344	.147	.175	2.34	-	.03*	.03	1.20
	Failure	.289	.131	.192	2.21	-	.02*	.02	1.60
Step 1									
	**Depression**	-	-	-	-	2.82	.28**	.39	-
Step 2									
	Social Isolation	.499	.130	.297	3.83	-	.15**	.18	1.13

	Self-Sacrifice	.367	.151	.192	2.44	-	.05*	.05	1.16
	Dependence	.276	.137	.162	2.02	-	.02*	.02	1.21
Step 1									
	**Anxiety**	-	-	-	-	2.95	.29**	.41	-
Step 2									
	Dependence	.370	.119	.262	3.11	-	.17**	.20	1.34
	Emotional Inhibition	.331	.136	.189	2.43	-	.04*	.04	1.15
	Defectiveness	.285	.125	.186	2.28	-	.03*	.03	1.70
Step 1									
	**Hostility**	-	-	-	-	2.73	.27**	.37	-
Step 2									
	Defectiveness	.400	.124	.254	3.22	-	.12**	.14	1.12
	Self-Sacrifice	.339	.130	.208	2.67	-	.06*	.06	1.14
	Emotional Inhibition	.287	.142	.159	2.02	-	.02*	.02	1.30
Step 1									
	**Phobic Anxiety**	-	-	-	-	1.10	.13	.15	-
Step 2									
	Defectiveness	.276	.120	.189	2.31	-	.06*	.06	1.07
	Emotional Inhibition	.314	.137	.188	2.30	-	.03*	.03	1.07
Step 1									
	**Paranoid ideation**	-	-	-	-	2.14	.23*	.30	-
Step 2									
	Dependence	.311	.135	.193	2.30	-	.10**	.11	1.24
	Mistrust/Abuse	.365	.138	.214	2.64	-	.05*	.05	1.16
	Enmeshment	.210	.106	.162	1.99	-	.02*	.02	1.18
Step 1									
	**Psychoticism**	-	-	-	-	3.34	.31**	.45	-
Step 2									
	Punitiveness	.326	.096	.275	3.38	-	.18**	.22	1.31
	Defectiveness	.272	.098	.224	2.77	-	.05*	.05	1.29
	Emotional Inhibition	.254	.104	.184	2.45	-	.03*	.03	1.11

Note. *= Correlation is significant at the 0.01 level (2-tailed). **= Correlation is significant at the 0.05 level (2-tailed). YSQ = Young Schema Questionnaire. *B*= Unstandardised Coefficient, SE=Standard Error, β=Standardised Beta Coefficient, R^2=R squared, f^2 = Cohens *f*. GSI = Global Severity Index. VIF = Variance Inflation Factor where a VIF of 1 = No correlation, < 3 = low correlation, > 5 = high correlation).

Supplementary Table S9
Regression Analysis for Pre-Treatment YSQ Change and Symptom Change for Each YSQ Version and DASS-42 Subscales in the Alcohol and Substance Use Group

				**YSQ-R**					
DASS-42 Subscales		*B*	SE	β	*t*	*F*	R^2 (Change)	f^2	VIF
Step 1									
	**Total Score**	-	-	-	-	1.66	.18**	.22	-
Step 2									
	Vulnerability to Harm	.054	.022	.199	.054	-	.05*	.05	1.17
	Punitiveness (Other)	-.226	.072	-.248	-.23	-	.04*	.04	1.14
	Insufficient Self Control	.073	.029	.206	.073	-	.03*	.03	1.25
Step 1									
	**Depression**	-	-	-	-	1.93	.21**	.27	-
Step 2									
	Vulnerability to Harm	.096	.025	.288	3.82	-	.05*	.05	1.06
	Punitiveness (Other)	-.275	.084	-.247	-3.27	-	.06**	.06	1.06
Step 1									
	**Anxiety**	-	-	-	-	1.33	.15	.18	-
Step 2									
	Insufficient Self Control	.099	.028	.275	3.47	-	.05*	.05	1.12
	Punitiveness (Other)	-.174	.074	-.187	-2.37	-	.08**	.09	1.12
Step 1									
	**Stress**	-	-	-	-	1.27	.15	.18	-
Step 2									
	Failure	.036	.014	.192	2.53	-	.04*	.04	1.00
				**YSQ-L3**					
DASS-42 Subscales		*B*	SE	β	*t*	*F*	R^2 (Change)	f^2	VIF
Step 1									
	**Total Score**	-	-	-	-	2.17	.21*	.27	-
Step 2									
	Vulnerability to Harm	.062	.021	.223	2.96	-	.05*	.05	1.00
Step 1									
	**Depression**	-	-	-	-	2.15	.21*	.27	-
Step 2									
	Vulnerability to Harm	.063	.035	.186	1.81	-	.05*	.05	1.98
	Punitiveness	-.435	.121	-.376	3.59	-	.03*	.03	2.07
	Self-Sacrifice	.093	.045	.168	2.08	-	.03*	.03	1.24
	Negativity	.128	.063	.258	2.05	-	.02*	.02	3.00
Step 1									
	**Anxiety**	-	-	-	-	1.96	.19*	.23	-
Step 2									
	Subjugation	.050	.19	.197	2.59	-	.03*	.03	1.00
Step 1									
	**Stress**	-	-	-	-	1.76	.18*	.22	-
Step 2									
	Negativity	.102	.034	.228	.230	3.03	.05*	.05	1.00
				**YSQ-S3**					
DASS-42 Subscales		*B*	SE	β	*t*	*F*	R^2 (Change)	f^2	VIF
Step 1									
	**Total Score**	-	-	-	-	1.33	.14	.16	-
Step 2									
	Negativity	.092	.031	.222	2.94	-	.05*	.05	1.00
Step 1									
	**Depression**	-	-	-	-	1.53	.16	.19	-
Step 2									
	Vulnerability to Harm	.069	.025	.207	2.73	-	.04*	.04	1.00
Step 1									
	**Anxiety**	-	-	-	-	1.24	.13	.15	-
Step 2									
	Vulnerability to Harm	.051	.021	.182	2.39	-	.03*	.03	1.00
Step 1									
	**Stress**	-	-	-	-	1.14	.12	.14	-

Step 2								
Negativity	.112	.034	.245	3.27	-	.06*	.06	1.00

Note. *= Correlation is significant at the 0.01 level (2-tailed). **= Correlation is significant at the 0.05 level (2-tailed). YSQ = Young Schema Questionnaire. DASS = Depression, Anxiety, and Stress Scale, *B*= Unstandardised Coefficient, SE=Standard Error, β=Standardised Beta Coefficient, R^2=R squared, f^2 = Cohens *f*. GSI = Global Severity Index. VIF = Variance Inflation Factor.
